# Salinity Duration Differently Modulates Physiological Parameters and Metabolites Profile in Roots of Two Contrasting Barley Genotypes

**DOI:** 10.3390/plants10020307

**Published:** 2021-02-05

**Authors:** Emilia Dell’Aversana, Kamel Hessini, Selma Ferchichi, Giovanna Marta Fusco, Pasqualina Woodrow, Loredana F. Ciarmiello, Chedly Abdelly, Petronia Carillo

**Affiliations:** 1 Department of Enviromental Biological and Pharmaceutical Sciences and Technologies, University of Campania “Luigi Vanvitelli”, Via Vivaldi 43, 81100 Caserta, Italy; emiliadellaversana@gmail.com (E.D.); giovannamarta.fusco@unicampania.it (G.M.F.); pasqualina.woodrow@unicampania.it (P.W.); loredanafilomena.ciarmiello@unicampania.it (L.F.C.); 2 Department of Biology, College of Sciences, Taif University, P.O. Box 11099, Taif 21944, Saudi Arabia; k.youssef@tu.edu.sa; 3 Laboratory of Extremophile Plants, Center of Biotechnology of Borj Cedria, University of Elmanar, Tunis 1068, Tunisia; selmaferchichi1@gmail.com (S.F.); chedly.abdelly@cbbc.rnrt.tn (C.A.)

**Keywords:** wild barley, osmotic adjustment, osmolality, potassium to sodium ratio, proline, asparagine, GABA

## Abstract

*Hordeum maritimum* With. is a wild salt tolerant cereal present in the saline depressions of the Eastern Tunisia, where it significantly contributes to the annual biomass production. In a previous study on shoot tissues it was shown that this species withstands with high salinity at the seedling stage restricting the sodium entry into shoot and modulating over time the leaf synthesis of organic osmolytes for osmotic adjustment. However, the tolerance strategy mechanisms of this plant at root level have not yet been investigated. The current research aimed at elucidating the morphological, physiological and biochemical changes occurring at root level in *H. maritimum* and in the salt sensitive cultivar *Hordeum vulgare* L. cv. Lamsi during five-weeks extended salinity (200 mM NaCl), salt removal after two weeks of salinity and non-salt control. *H. maritimum* since the first phases of salinity was able to compartmentalize higher amounts of sodium in the roots compared to the other cultivar, avoiding transferring it to shoot and impairing photosynthetic metabolism. This allowed the roots of wild plants to receive recent photosynthates from leaves, gaining from them energy and carbon skeletons to compartmentalize toxic ions in the vacuoles, synthesize and accumulate organic osmolytes, control ion and water homeostasis and re-establish the ability of root to grow. *H. vulgare* was also able to accumulate compatible osmolytes but only in the first weeks of salinity, while soon after the roots stopped up taking potassium and growing. In the last week of salinity stress, the wild species further increased the root to shoot ratio to enhance the root retention of toxic ions and consequently delaying the damages both to shoot and root. This delay of few weeks in showing the symptoms of stress may be pivotal for enabling the survival of the wild species when soil salinity is transient and not permanent.

## 1. Introduction

In the Mediterranean basin, salt increase in soils is favored by the hot and dry climate in the spring- summer period, high rate of evapotranspiration, low rainfalls and recurrent seawater intrusions in aquifers [1,2]. Moreover, low rainy autumn-winter seasons increase salinity by causing damage to both irrigated spring/summer crops and non-irrigated winter crops [1]. In the future, the salt related desertification could cause the loss of thousands of hectares further reducing agricultural soil availability and yield [3]. Unfortunately, this is a very common occurrence. In fact, around 20% of irrigated land worldwide providing one-third of food is affected by salt [4]. Salinity limits plant growth and development by disturbing ions and water uptake, affecting nitrogen metabolism and causing oxidative stress [5,6]. The detrimental impact on plant performance depends on the stage of application, plant species and salinity dose and duration among other [7,8]. Increasing salinity leads to a high concentration of rhizospheric ions (mainly Na^+^ and Cl^−^), thus causing a significant depletion in water potential. Therefore, salinity has a dual impact on plant performance, acting either as an inhibitor of water uptake by roots, via an osmotic effect or as an accumulator of Na^+^ and Cl^−^ ions, with subsequent toxic impacts [9,10,11]. Roots are the first organ of plant to sense salinity in the rhizosphere and are the initial site to suffer from salt stress. Salinity reduce root development by inhibiting both root cell production and expansion and limiting the length of mature epidermal cells. These effects could be due to the toxicity of salts on the expanding cells metabolism, the reduced water availability for cell expansion and the induction of plant responses [12,13,14].

The superiority of tolerant species, as compared to sensitive ones, to cope with salt stress is associated with a better tolerance mechanisms [9,15] and the activation and regulation of several specific stress-related genes [16,17,18]. The reduction in growth rate is lower in plants with a high ability to exclude Na^+^ and Cl^−^ by preventing their entry into the vascular system, in this way the concentrations of these toxic ions are kept low in the shoot [9,19,20]. A ubiquitous mechanism used by plants to limit the salt stress damage is the compartmentalization of salts (mainly Na^+^ and Cl^−^) into vacuoles and the synthesis of compatible osmolytes, which do not interfere with cellular metabolism even at high concentration, for cytoplasmic osmoregulation [21,22,23]. The main compatible osmolytes observed in various plant systems are low molecular weight nitrogen-containing compounds such as amino acids, amines and betaines and sugars [24,25,26]. They are responsible for osmotic adjustment and oxidative stress protection in the cytosol [10,27,28].

Cereals cultivation is widespread all over the world and salinity is among the abiotic stresses that mostly limit their growth. They can be sorted according to their degree of sensitivity to salt in the following decreasing sense: rice (*Oryza sativa* L.), durum wheat (*Triticum durum* L.), bread wheat (*Triticum aestivum* L.) and barley (*Hordeum vulgare* L.) [29]. Looking for a valid solution to cope with salinity is essential because cereals provide more than 50% of a human energy and protein needs [30]. Many strategies have been adopted to limit damages induced by salt stress to crops. Genetics and genomics studies have allowed the identification and functional characterization of genes/QTLs responsible for salinity tolerance, then utilized for crop improvement through genetic engineering or marker assisted breeding or a combination of both [31,32,33,34]. However, crop tolerance improvement by transgenic technology is not easily accessible to researchers and farmers, because it is expensive, time consuming and still debated in most countries due to ethical and political issues [35]. More easily adaptable and cost-effective approaches to cope with salinity involve agronomic practices. Variety selection and the use of soil conditioners and fertilizers, and/or application of biostimulants to seeds, seedlings or plants before exposure to salinity can make plants facing salt stress more easily [35,36,37,38]. In addition, leaching still is the main mean to support salinity management but this practice is impossible to apply in arid areas [39]. Therefore, domesticating halophytes or bringing relevant tolerance traits to salt sensitive crop species is one of the most useful approach in the reclamation of degraded soils and can open the way to use salt-affected and salinized territories [7,15,40,41].

Barley is the most-tolerant cereal but it cannot survive under salinity higher than 250 mM NaCl persisting for a long period [8,42]. In addition, wild barley species are more salt tolerant than cultivated barley (*Hordeum vulgare* L.) [43]. Saline habitats are occupied by more than half of the wild *Hordeum* species. However, data on their tolerance mechanisms, useful to improve the tolerance of the related crop species, are largely unexplored [44]. Furthermore, the complexity of the salt tolerance character together with the insufficient knowledge of the physiological and biochemical behavior of these plants, in particular at root level, are the main factors preventing the development of salt tolerant cultivars. Moreover, it would be of considerable interest to consider the physiological responses of plants when the high salts concentration in the growth medium is not a permanent condition by assessing their ability to recover from stress. Ferchichi et al. [43] have already shown the different temporal accumulation pattern of leaf metabolites that allow *Hordeum vulgare* cv. Lamsi, a cultivated barley and *Hordeum maritimum* With. (*H. marinum* Huds. subsp. marinum), a wild *Hordeum* species, to respond to extended salinity. This is a complementary study aimed to investigate the dissimilar root physiological responses to salt stress of the same two Tunisian species of barley. For this purpose, the seedlings of the two barley contrasting genotypes were subjected to hydroponic culture under (i) normal growth conditions without salt (control), (ii) salt stress with 200 mM NaCl in the nutrient solution (stress) and (iii) initial salt stress with 200 mM NaCl and then removal of stress and return to non-saline conditions (salt removal). Our main objective was to unravel the mechanisms which differentiate wild and cultivated barley roots in coping with salinity and recovering from salt stress by studying their physiological and biochemical responses in comparison with control plants.

## 2. Results

### 2.1. Growth and Physiological Parameters

**Figure 1:**
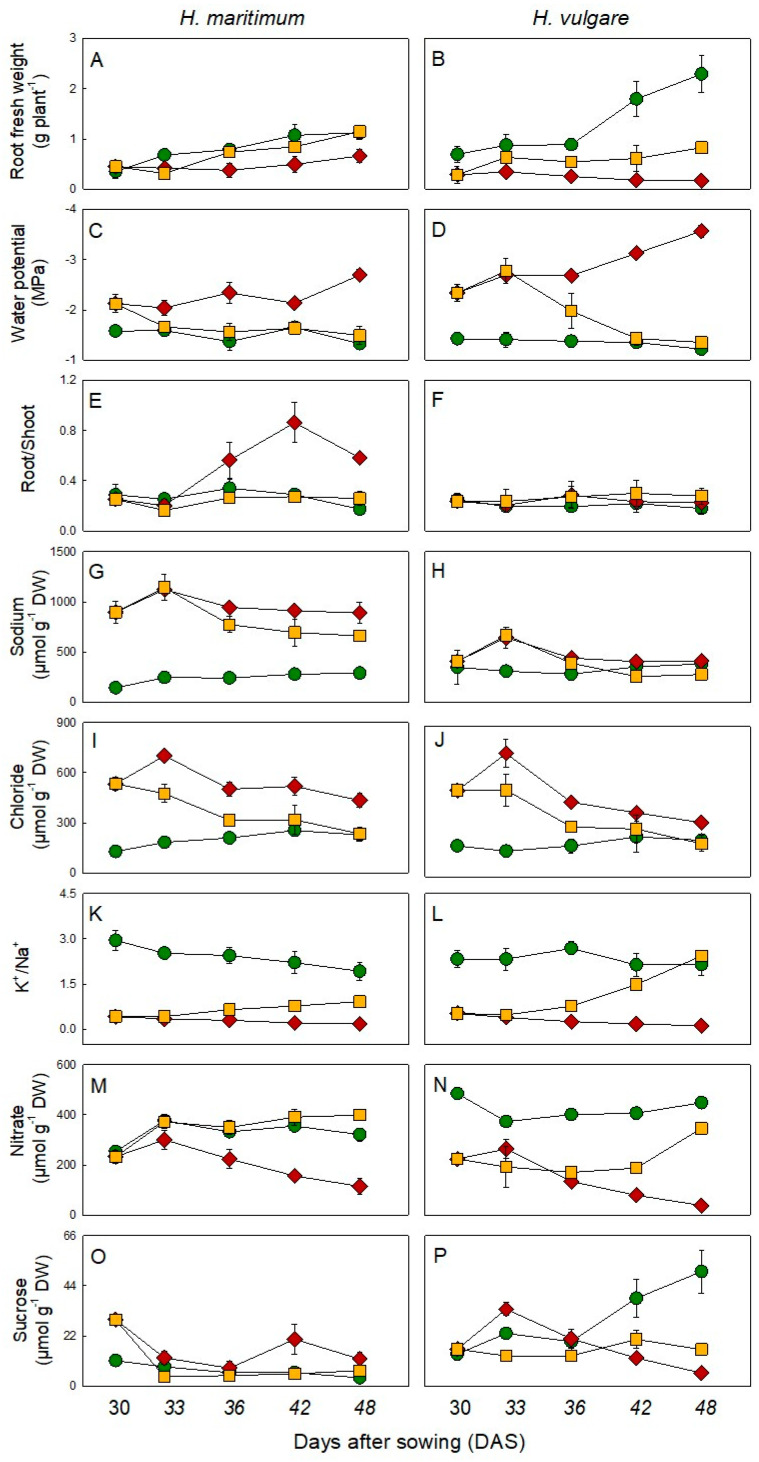
Main physiological parameters, inorganic ions and sucrose in roots of *H. maritimum* and *H. vulgare* under control (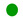), salt stress (200mM NaCl,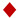) and salt removal treatments (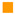). Salt was gradually added to salt treated plants starting from 15 days after sowing (DAS). Salt removal treatment started from 30 DAS. Harvests were conducted at 30, 33, 36, 42 and 48 DAS. Values are mean ± s.d. (*n* = 3).

The two barley species under control conditions showed an exponential expansion in the period 30–48 days after sowing (DAS) with the cultivated species presenting, on average, higher FW (+68%) than the wild one (Figure 1A,B). Salinity treatments severely inhibited roots growth in terms of fresh weight in both plant species, affecting more *H. vulgare* than *H. maritimum*, with the former showing a minimum average value at 42–48 DAS (12-fold lower than respective controls) (Figure 1B). The wild species showed a greater belowground development system in responses to salt stress development; this is evidenced by a higher root to shoot ratio, especially in the last days of the experiment (3.7- and 2.5-fold higher than the cultivated species at 42 and 48 DAS, respectively). However, these differences were not particularly evident under control conditions (Figure 1E). After removal of stress, only the belowground fresh weight system of *H. maritimum* could fully recover from salinity; on the contrary, *H. vulgare* roots biomass production was nearly threefold lower than the respective control plants (Figure 1E,F). The relative water content (RWC) of both control species was on average 90.4%, which remained constant in *H. maritimum*-stressed plants but decreased (–11%) in *H. vulgare* plants in the same condition at 36–48 DAS (Table S1). At 30 DAS, under control conditions, *H. vulgare* and *H. maritimum* showed water potential values of –1.42 and –1.58 MPa, respectively, which slightly increased at 48 DAS. Salt stress significantly decreased this parameter, which, at 48 DAS, resulted 2.0 and 2.9- fold lower in *H. maritimum* and *H. vulgare* plants in comparison with the respective controls (Figure 1C,D).

In *H. maritimum* and *H. vulgare* under salinity at 30 DAS the sodium concentration was 896 (6.3-fold higher than the respective control) and 409 μmol g^−1^ DW, respectively. The sodium concentration underwent a statistically significant change (*p* < 0.05) at 33 DAS in both species.

At 48 DAS the salt removal treatments showed sodium concentrations equal to 661 and 277 μmol g^−1^ DW in the wild and cultivated species, respectively (Figure 1I,J; Table S1). Chloride concentration was on average 198 μmol g^−1^ DW in control plants of both species throughout the experiment. It increased in both plant species under salinity, showing an average concentration of 525 μmol g^−1^ DW at 30 DAS, which reached its maximum value at 33 DAS, with on averaged value of 718 μmol g^−1^ DW. While, at 42–48 DAS, chloride was on average 34.5% higher in *H. maritimum* than in *H. vulgare*. In the recovery treatment chloride content at the end of the experiment did not significantly differ from the respective controls (Figure 1). The initial level of potassium was 423 and 814 μmol g^−1^ DW in *H. maritimum* and *H. vulgare* control plants, respectively. Salt stress significantly decreased the concentration of this ion in *H. vulgare* even at 30 DAS. It soon after shortly increased at 33 DAS and then started strongly decreasing (Table S1). This ion started significantly decreasing in Hordeum maritimum since 36 DAS. At the end of the experiment, the potassium reduction was more marked in *H. vulgare* than in *H. maritimum*, resulting 17.8- and 3.5-fold lower than controls, respectively (Table S1). Removal of stress significantly increased potassium level, which, starting from 36 DAS, got concentrations in both species that did not significantly differ from the respective controls (Table S1). The ratio of potassium to sodium content, used for understanding plants’ ability to discriminate the two ions [45], did not significantly differ in control plants of both species (on average 2.37 throughout the experiment). However, it strongly decreased in stressed plants, showing at 48 DAS values of 0.18 and 0.11 in *H. maritimum* and *H. vulgare,* respectively, 10.7 and 19.6-fold lower than respective controls (Figure 1K,L; Table S1). The removal of salt stress determined an increase in potassium to sodium ratio in both species; however, while in *H. vulgare* the value of this ratio at 48 DAS matched that of control (Figure 1L), in *H. maritimum* the recovery just got a value 2.1- fold lower than its control (Figure 1K). The initial nitrate concentration in *H. maritimum* control and stressed plants was on average 243 μmol g^−1^ DW; however, in the latter condition a constant reduction was observed towards the end of the treatment. *H. vulgare* control plants initially accumulated a concentration of nitrate 1.91-fold higher than wild species but salinity strongly decreased it until a final value at 48 DAS of 38 μmol g^−1^ DW, that was 3-fold lower than that in *H. maritimum* stressed treatment. At 48 DAS, the nitrate concentration in roots of both species was reestablished nearly to control ones after the stress was relieved (Figure 1M,N).

Salinity causes the generation of reactive oxygen species (ROS) within plant cells and MDA and H_2_O_2_ are used as markers to evaluate the extent of oxidative damage [46]. Under control conditions, the MDA content at 30 DAS in both species was on average 36.4 nmol g^−1^ DW. In *H. Maritimum* it decreased at the end of the experiment (42–48 DAS), while an opposite trend was observed in *H. vulgare*. In presence of salinity, the concentration of MDA was constantly lower than that in control of the wild species, while in the cultivated one it increased at 33 DAS resulting lower in the following days (Table S1). The H_2_O_2_ content showed a similar trend to that of MDA in *H. vulgare* under both experimental conditions. Salinity decreased it in *H. maritimum* in the period 33–42 DAS. In the salt removal condition, the MDA and H_2_O_2_ increased getting values comparable to those of respective controls (Table S1).

### 2.2. Carbohydrates Content

Starch content in *H. maritimum* and *H. vulgare* control plants at 30 DAS was 88.4 and 148 µmol glucose equivalents (G_eq_) g^−1^ DW. This value decreased at 42 and 48 DAS in the wild species (–30% and 39%, respectively), while it remained unchanged in the cultivated one (Table S1). Under salinity, starch content in *H. maritimum* and *H. vulgare* was initially 126 and 81 µmol G_eq_ g^−1^ DW, 42.5% higher and 45.3% lower than controls, respectively. In the following days, this value decreased in the wild species without undergoing statistically significant variations compared with respective controls. However, *H. vulgare* stressed plants showed a starch content lower than respective controls for all the experimental period. After the removal of stress, the content of starch in both species got values similar to those of the respective controls (Table S1). In both species hexoses content showed the same behavior of that of starch, except for the samples of *H. vulgare* at 33 and 36 DAS under salinity, in which the content of these soluble sugars increased at the same values of the controls and the recovery treatment in which the content of hexoses increased until 48 DAS but remaining 40% lower than the respective control (Table S1). During the time course of experiment, under control conditions, sucrose concentration had a similar trend of hexoses decreasing in *H. maritimum* while increasing in *H. vulgare*. Under salinity, it increased in the wild species (except at 33 and 36 DAS) and decreased in the cultivated ones at 42 and 48 DAS, compared with respective controls. The salt removal determined at 48 DAS a sucrose concentration similar to that of control in the wild species, while it remained 70% lower in the cultivated one compared to that of the respective control (Figure 1O,P; Table S1).

### 2.3. Nitrogen Containing Compounds

**Figure 2:**
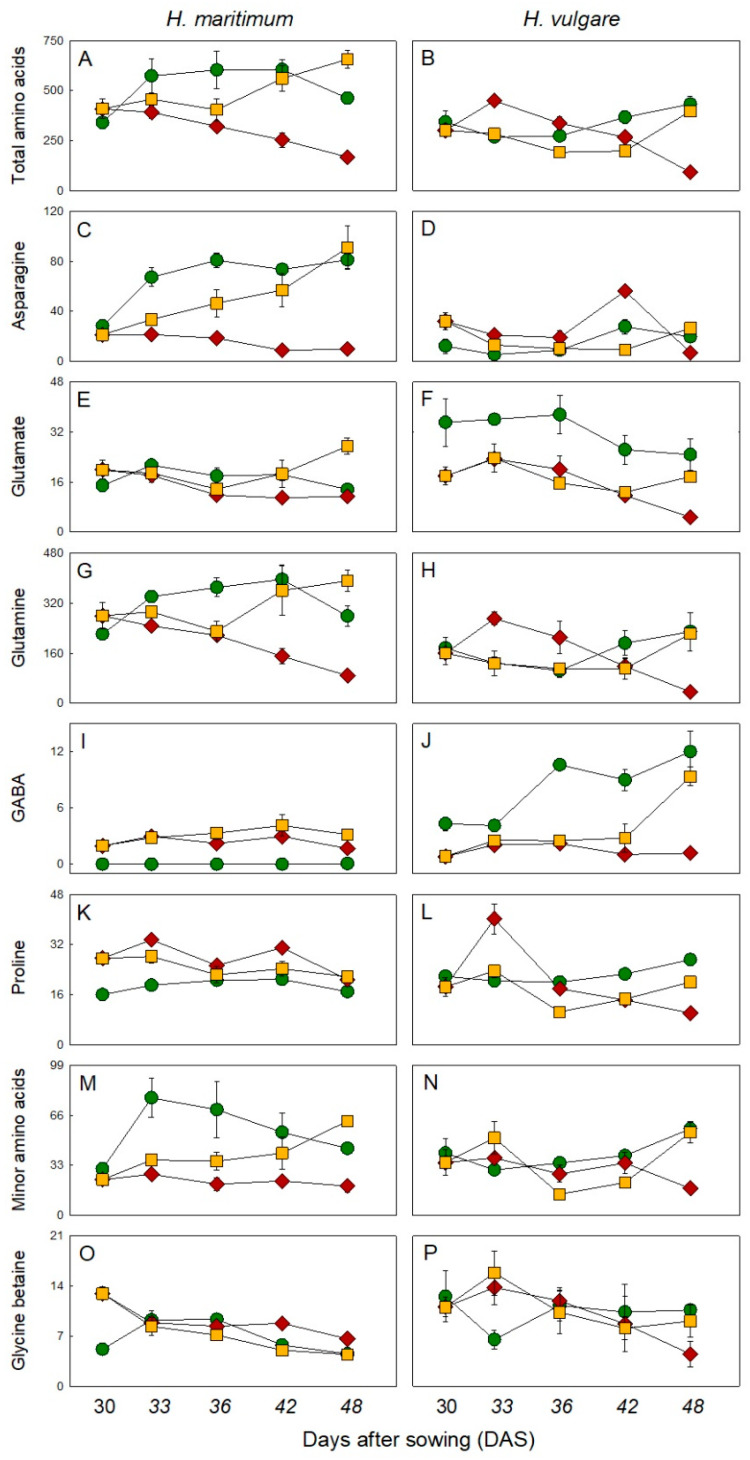
Amino acids and glycine betaine levels in roots of *H. maritimum* and *H. vulgare* under control (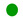), salt stress (200mM NaCl, 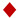) and salt removal treatments (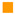). Salt was gradually added to salinity treatments starting from 15 days after sowing (DAS). Harvests were conducted at 30, 33, 36, 42 and 48 DAS. Metabolites were expressed as µmol g^–1^ DW. Values are mean ± s.d. (*n* = 3).

Under control conditions, at 30 DAS, the content of proteins in *H. maritimun* was 20.9 mg g^−1^ DW; starting from 33 DAS its value doubled, remaining constant until 48 DAS. In the cultivated species protein content at 30 DAS was 63 mg g^−1^ DW and in the period 36–48 DAS it further increased (+1.7-fold) (Table S1). Under salinity, on the contrary, the concentration of proteins in the two species was averagely higher in the wild species than in the cultivated one. In particular, at 42 and 48 DAS, the protein content in *H. vulgare* dropped to a value 1.5- and 6.3-fold lower than that of *H. maritimum* under the same treatment. The recovery treatments showed at 48 DAS a protein content similar to that of the respective controls (Table S1). The total free amino acids in both species had a similar initial concentration (on average 342 µmol g^−1^ DW). In *H. maritimum* at 33 DAS the content of protein increased of 1.7-fold compared to the initial value, then decreasing in the subsequent days of treatment (Figure 2A); while it did not undergo statistically significant differences in *H. vulgare* (Figure 2B). Glutamine was quantitatively the major amino acid of *H. maritimum* representing about 61% of its total amino acid content both in control and salt stressed samples, followed by asparagine, minor amino acids, glutamate and ornithine, which on average represented in the control treatment the 12, 9.1, 3.3 and 2.4% of the total content, respectively (Figure 2; Table S1). Glutamine, minor amino acids, glutamate, alanine and asparagine were the major amino acids of *H. vulgare* control plants representing, on average, 45.9, 10.8, 8.8, 5.5 and 4.1% of the total amino acid concentration (Figure 2; Table S1). It is important to underline that asparagine content was much more concentrated in the control treatment of wild species compared to the same treatment of the cultivated one, showing a content 12.1 and 9.0-fold higher than that of cultivated species at 33 and 36 DAS, respectively (Figure 2C,D). Glutamine content in *H. maritimum* was also 3.2, 3.5 and 2.1-fold higher than that of *H. vulgare* at 33, 36 and 42 DAS, respectively (Figure 2E,F). On the contrary, GABA content, which strongly varied in dependence on species and the experimental conditions, in control conditions was much higher in the cultivated species than in the wild one (Figure 2I,J).

Salt stress induced a change of root amino acid profile in both species (Figure 2, Table S1). In *H. maritimum* under salinity, asparagine, ornithine and minor amino acids steadily decreased while GABA increased. Aspartate and glutamate had initially a concentration higher than respective controls but they decreased from 33 DAS together with glutamine. In *H. vulgare* under salinity the majority of amino acids decreased compared with the respective controls. The contents of GABA, glutamate and ornithine were lower than those of control for the entire experimental period. Alanine and glycine concentration decreased from 36 DAS, while minor amino acids, which showed a concentration similar to that of control for almost all the experimental period, strongly decreased at 48 DAS. Initially, in the cultivated species, asparagine and glutamine increased and then decreased towards the end of the experiment. The content of aspartate showed a fluctuating trend resulting 2.6-fold lower than respective control at 48 DAS. An opposite trend was observed for proline content, moreover salt stress increased it in both species being, however, on average doubled in *H. vulgare* compared with *H. maritimum.* For the majority of amino acids, the removal of salt stress cancelled the impact of the first phase of salt treatment (15 days) and corrected their levels to nearly the control ones in the cultivated species, while some of them significantly increased in the wild species compared to control. In particular, the amino acids that at 48 DAS of the recovery treatment strongly increased in *H. maritimum* compared to the respective control were alanine (+2.9-fold), aspartate (+2.2-fold), GABA (+43.7-fold), glutamine (+1.4-fold) and minor amino acids (+1.4-fold) (Figure 2; Table S1). *H. vulgare* control plants had, on average, a higher glycine betaine (GB) concentration (+51.4%) than *H. maritimum* and was not particularly influenced by salinity except at 33 and 48 DAS being 2.1-fold higher and 2.3-fold lower than respective controls. Salt stress increased its content in *H. maritimum* at 30, 42 and 48 DAS (Figure 2O,P).

### 2.4. Sap Osmolality and Contribution of Ions and Metabolites to the Osmotic Adjustment

Sap osmolality was 330 and 354 mOsmol kg^−1^ in the control plants of *H. maritimum* and *H. vulgare*, respectively. In the presence of salinity at 30 DAS, it significantly increased (*p* < 0.05) only in the wild species (+1.9-fold) (Table 1A; Table S1). However, at the end of the experimental period, both species showed sap osmolality values higher than controls (+2.0 and 1.6-fold in the wild and cultivated species, respectively). The removal of salt stress completely restored the control values of sap osmolality in the cultivated species since 33 DAS, while in the wild species it remained higher than control even after 18 days of recovery (+1.4-fold) (Table 1A; Table S1).

The relative contribution of the inorganic ions to osmolality at 30 DAS was, on average, 39.5% independently of species and treatments. In particular, the main contribution came from potassium and nitrate in control samples and from sodium and chloride in salt stressed samples. In particular, the contribution of chloride was higher in *H. vulgare* under salinity. In the wild species under salinity at 36 and 48 DAS, sodium contribution to osmolality did not differ from that at 30 DAS, while in the cultivated one it strongly increased reaching the value of 15.6% and 17.3%, 1.3 and 1.5-fold higher than that at 30 DAS respectively. The contribution of the measured organic osmolytes to osmolality under control conditions at 30 DAS was 19.3 and 10.3% in *H. maritimum* and *H. vulgare,* respectively; while it decreased (–40%) in the wild stressed plants. While at 36 DAS under salinity, the measured organic osmolytes contribution in *H. maritimum* slightly increased, at 48 DAS it was lower than that at 30 DAS (–59%). In *H. vulgare,* it initially increased (+71%) at 36 DAS and then showed a strong decrease (–59%) at 48 DAS compared to the initial value (Table 1B). Since only a part of the drop in osmotic potential can be explained by organic solutes, with a lesser degree of inorganic ones, we also assume that other composites, such as organic acids and dimethylsulphniopropionate (DMSH), may also play non negligible role in the osmotic adjustment of both barley genotypes [22,47].

### 2.5. Principal Component Analysis

**Figure 3:**
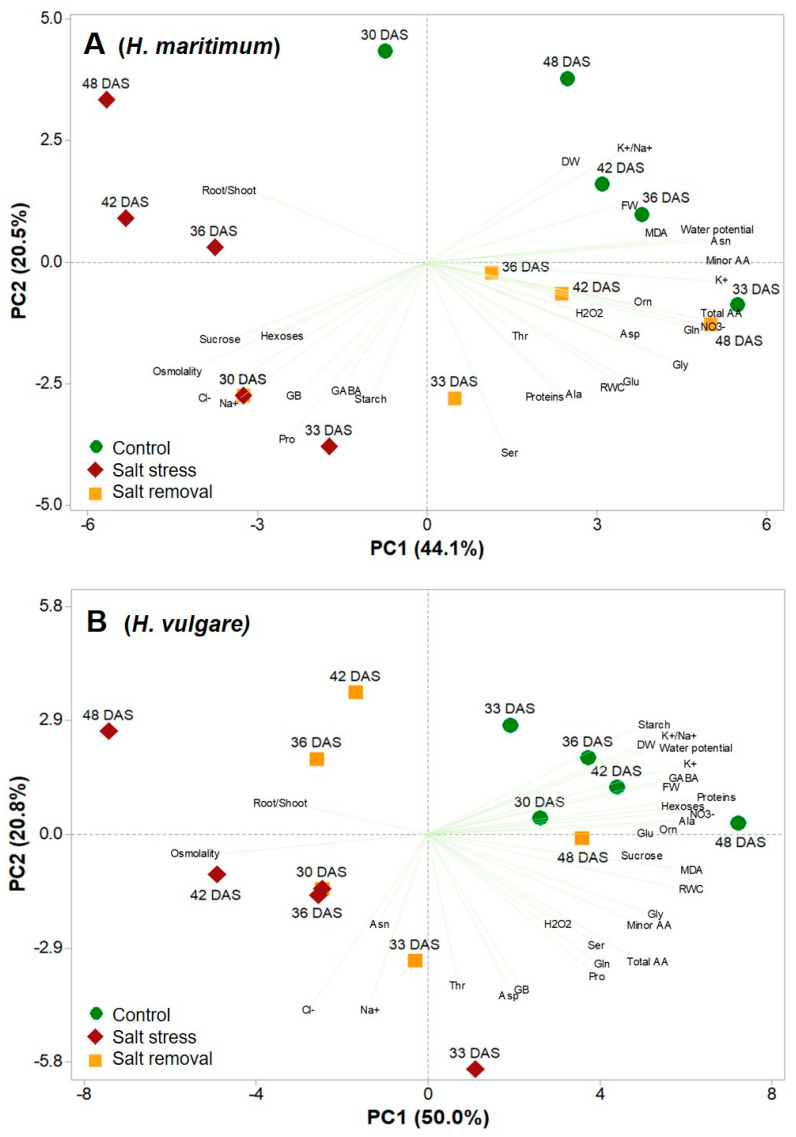
Principal component loading plot and scores of principal component analysis (PCA) of physiological and biochemical parameters of roots of *H. maritimum* (**A**) and *H. vulgare* (**B**) under control, salt stress (200mM NaCl) and salt removal treatments. Salt was gradually added to salinity treatments starting from 15 days after sowing (DAS). Harvests were conducted at 30, 33, 36, 42 and 48 DAS.

To obtain an overview on the morphological and biochemical parameters characterizing the two species during the long-lasting salt and salt removal treatments compared to controls, a principal component analysis (PCA) was carried out for *H. maritimum* (Figure 3A) and *H. vulgare* (Figure 3B). The analysis showed that for both species, the first four principal components (PCs) were associated with Eigen values higher than 1 and explained 89.6% of the cumulative variance, with PC1, PC2, PC3 and PC4 accounting for 44.1%, 20.5%, 9.1% and 5.1% respectively for *H. maritimum* (Figure 3A) and 83.9% of the cumulative variance, with PC1, PC2, PC3 and PC4 accounting for 50.0%. 20.8%, 7.4% and 5.7% respectively for *H. vulgare* (Figure 3B). In the wild species, PC1 was positively correlated to potassium, asparagine, minor and total amino acids, nitrate, water potential, glutamine, glycine and MDA. PC1 was also negatively correlated to osmolality, chloride, sucrose, sodium, root to shoot ratio and hexoses. Moreover, PC2 was positively correlated to potassium to sodium ratio, DW, root to shoot ratio and FW; and negatively correlated to serine, proline, starch, proteins, glycine betaine, alanine, GABA, RWC and glutamate. Furthermore, the score plot of the PCA divided the three treatments along PC1 with the control having the highest total and minor amino acid content, water potential, asparagine, glutamine, potassium, potassium to sodium ratio and nitrate in the positive side, for the most in the upper right quadrant; and salt removal treatment with the highest proteins, glutamate, alanine, RWC, glycine, aspartate, H_2_O_2_ and threonine in the lower right quadrant of the positive side, except at 30DAS. The salt stress treatment on the negative side, was present both in the lower and upper left quadrant, clustering with osmolality, chloride, sucrose, sodium, hexoses, glycine betaine, proline, GABA and starch (30–33 DAS) and with root to shoot ratio (36–48 DAS), respectively (Figure 3A).

In the cultivated species, PC1 was positively correlated to proteins, nitrate, potassium, alanine, MDA, RWC, GABA, hexoses, ornithine, FW, potassium to sodium ratio, water potential, glycine, starch and DW; while it was negatively correlated to osmolality and root to shoot ratio. Moreover, PC2 was positively correlated to starch and negatively correlated to chloride, sodium, aspartate, threonine, glycine betaine, proline, glutamine, total and minor amino acids, serine and asparagine. Furthermore, the score plot of the PCA divided also the three treatments of *H. vulgare* along PC1 with the controls having the highest hexoses, DW, FW, sucrose, GABA, alanine, proteins, nitrate, potassium e potassium to sodium ratio in the positive side, concentrated in the upper right quadrant. The salt stress treatments on the negative side, were present both in the lower and upper left quadrant, except 33 DAS that was in the lower right quadrant in the most negative side of PC2 close to *y*-axis. This latter clustered with sodium and chloride, aspartate, glycine betaine, proline, threonine, total amino acids, glutamine and serine; while the other salt stress treatments clustered with osmolality and with root to shoot ratio. The salt removal treatment at 33 DAS showed a similar clustering pattern of salinity at 33 DAS. Besides, 36 and 42 DAS were in the upper left quadrant clustered with root to shoot ratio, while 48 DAS was in the lower right quadrant close to *x*-axis, clustered with glycine, MDA, RWC, threonine and serine (Figure 3B).

## 3. Discussion

Plant responses to salinity, which depend not only on species but also on concentration of salts and salt exposure duration, entail specific plant physiological, anatomical and molecular changes [8,11,43,48]. Roots, which are in direct contact with the saline solution of the soil, are the first organs to suffer from the damages caused by high salinity and this adversely impacts growth and development of most plant species [49,50]. The salts cause a decrease of water potential in the root-growing medium, lowering the plant capacity to uptake water and nutrients. Moreover, the high concentration of toxic ions in the root cells usually induces oxidative stress and ROS formation [34], inhibiting root cell division and elongation [50,51] and negatively influencing whole plant performance [52]. Given the essential role of roots to ensure the survival of plant, the comprehension of the mechanisms involved in the initiation of tolerance responses in salt tolerant vs. salt sensitive species is of pivotal importance. Therefore, in the present study, we compared plants of *H. maritimum* and *H. vulgare* under control, salt stress and salt removal treatments for 18 days after an initial period of 15 days of 200 mM NaCl salinity. Both species showed an exponential expansion during the period of study showing similar root to shoot ratios. However, as previously reported in Ferchichi et al. [43], *H. vulgare* was able to produce almost double the biomass of *H. maritimum* under control conditions, as a result of its higher photosynthetic yield (higher chlorophyll a and chlorophyll a/b ratio). This superiority of biomass accumulation was associated also with higher values of RWC, nitrate, potassium, proteins and starch content compared to wild species. On the contrary, plants of *H. maritimum* at 30 DAS, under control conditions, showed a series of basic responses that were very similar to the responses of the cultivated species under stress, probably because they were already constitutively expressed. In particular, these plants showed lower contents of potassium, starch and an unusual high asparagine content. Keys [53] suggested that the accumulation of asparagine under salinity could depend on amides transported to roots from leaves where they accumulate due to the re-assimilation of ammonia produced by photorespiration. In fact, this can be the consequence of the reduction of internal CO_2_ concentration and the increase of the Rubisco oxygenase activity caused by osmotic stress [54]. However, this was certainly not the case of the wild barley roots under control condition. It is possible that the high ammonia availability due to the cultivation in a culture media activated a defense response. In fact, it has been recently proved in *Salicornia europaea* that the addition of NH_4_Cl to the growth medium in the absence of NaCl reduced plant hydration, plant growth and caused tissue browning and tip burns. This was attributed to the metabolic and growth impairment caused by the lack of sodium that is the main osmoregulator in *Salicornia* as well as in the most of halophytes rather than to specific ammonium toxicity [55]. However, we found that potassium, which could partially replace the osmoregulatory functions of sodium, showed a concentration in *H. maritimum* that was half of that present in *H. vulgare* under control conditions at 30 DAS probably because of the competition effects of potassium with ammonium [55]. Therefore, the accumulation of asparagine and glutamine in this halophyte plant may contribute to osmotically regulate roots cells, building up the turgor potential for allowing extension growth when sodium and chloride concentrations are low and decreasing the cell water potential to uptake water and ions as suggested by Rozema, et al. [56].

Salinity treatment severely inhibited root fresh weight in both plant species, affecting it sooner (since 30 DAS) and more in *H. vulgare* than *H. maritimum* (Figure S1A,B). The decrease of root biomass in the cultivated species occurred simultaneously with a strong increase of chloride and a decrease of potassium to sodium ratio and nitrate. The metabolites pattern was completely altered, with starch, hexoses and proteins which decreased while asparagine and proline increased [6]. Indeed, starch reserves were laid down to provide carbon skeletons for the synthesis of metabolites that could take part in osmotic adjustment reducing the cytosol water potential and allowing leaf cell expansion under salt stress [57]. At 33 DAS, the concentration of compatible osmolytes, sucrose, amides, proline and GABA further increased in the cultivated species increasing the RWC and appearing able to maintain the intra-cellular osmotic balance between the cytoplasm and the sodium and chloride compartmentalized in vacuoles [26,58]. However, from 36 DAS, when the concentration of sodium strongly increased in shoots [43], root fresh weight, RWC, potassium to sodium ratio, nitrate, carbohydrates, proteins and amino acids content started decreasing getting values at the end of the experiment (48 DAS) much lower than those of wild species. These results suggest that, in cultivated species, the scarce ability of *H. vulgare* root cells to block the Na^+^ flux towards the shoot led to a reduction in photosynthetic efficiency and a consequent decrease of photosynthates exported to root, thus determining an incapacity of this organ to sustain the synthesis of expensive compatible solutes and causing the onset of root cell damage symptoms [50].

*H. maritimum*, after 15 days of salt stress (30 DAS), showed a greater belowground development system (Figure S1A) and higher root to shoot ratios compared to the cultivated species, notwithstanding it accumulated in roots not only similar amounts of chloride but also much higher amounts of sodium without being strongly affected. This great ability of *H. maritimum* to tolerate higher amounts of toxic ions and present fewer symptoms of toxicity was previously reported by Yousfi, et al. [59]. Commonly, the enzymes of salt-tolerant plants are not more tolerant to toxic inorganic ions than those of salt-sensitive ones [14]. However, salinity tolerance can be exerted by lowering toxic ions cytoplasmic levels. This wild barley species, in fact, is able to compartmentalize Na^+^ in the roots and restrict its xylem loading to shoot when exposed to 200 mM NaCl for more than 15 days [43]. Accordingly, Garthwaite, von Bothmer and Colmer [44] showed that seven wild *Hordeum* species had a better ability to exclude sodium from their leaves than cultivated barley when grown under different NaCl concentrations. The ability to exclude Na^+^ from the leaves is more pronounced in bread wheat compared with barley; nevertheless, wild barley has a more effective mechanism to compartmentalize toxic ions into vacuoles minimizing their damage and accumulating organic compounds to osmotically balance the cytosol, as proved by the higher sodium concentration in the roots [44]. A tonoplast Na^+^/H^+^ antiporter, energized by the V-type H^+^-ATPase and H^+^-PPase, guides the entry of sodium into the vacuole by limiting its harmful effects in the cytoplasm and favoring the osmotic adjustment [60]. In many plant systems the activity of pumps increases following exposure to high salinity, as shown in tonoplast vescicles from barley roots [61]. Indeed, in *H. maritimum* under salinity at 30 DAS, the restriction of sodium transport from roots to shoots increased tolerance to salinity allowing the photosynthetic apparatus to be preserved. Thus, recent photosynthates continued to be exported and accumulated into the roots as sucrose, hexoses and starch, contributing to supply carbon skeletons and energy for the synthesis of compatible metabolites. As consequence, in the roots of the wild species at 30 DAS the concentration of proline, GABA and glycine betaine increased, supporting a high RWC, protein synthesis and growth, notwithstanding the decrease of nitrate and potassium [5,26]. This allowed *H. maritimum* stressed plants to increase sap osmolality, suggesting the development of an active osmotic adjustment [22,62]. Moreover, glycine betaine, proline and GABA, as well as behaving as osmolytes, could act as ROS scavengers stabilizing and protecting macromolecules and membranes [26,58]. Even under prolonged salinity, when the roots of *H. maritimum* contained a double Na^+^ concentration compared with *H. vulgare*, the wild species managed to accumulate a greater K^+^ amount until the end of the experiment, notwithstanding the strong competition between Na^+^ and K^+^ for transport into the cell [63]. Moreover, the wild species, keeping low the cytoplasmic sodium concentration by increasing its accumulation in the vacuole, limited the membrane depolarization and improved the potassium uptake and retention [64]. In this regard, Ligaba and Katsuhara [65] showed that Na^+^/H^+^ antiporters gene expression (*HvNHX1*, *HvNHX3* and *HvNHX4*) was higher in roots of K305 (*H. vulgare* L. salt-tolerant cultivar) than in I743 (*H. vulgare* L. salt-sensitive cultivar) with prolonged exposure to salt, suggesting that this could represent a salt stress tolerance mechanism. In fact, this is very important since potassium is involved in a large number of developmental and physiological processes, including the regulation of cytosol pH and cell volume, activation of enzymes, thylakoid stacking, electron transport properties, stomatal movements and eventually photosynthetic efficiency [66,67]. At 42–48 DAS (27–33 days of exposure to 200 mM NaCl salinity), the roots of *H. maritimum* maintained higher concentrations of compatible osmolytes such as sucrose, glutamate, glutamine, proline and GABA compared to the cultivated species and further increased their tolerance to water salinity implementing the root to shoot ratio [68]. The strategy of increasing the root to shoot ratio allowed plants to confine the toxic ions within the roots by preventing their translocation to the aerial parts [11,48], maintaining still active the photosynthesis and the capacity to export recent photosynthates to roots. Therefore, the roots of wild species could still have carbon skeletons and energy available to sustain the GS-GOGAT pathway leading to the production of glutamine, glutamate, proline and proteins. However, this status could not be taken for much longer since the constant decrease of potassium, potassium to sodium ratio and nitrate demonstrate that *H. maritimum* had only a delay in showing the symptoms of stress and damage in comparison with *H. vulgare* (Figure 3A,B) as also found in Ferchichi, et al. [43].

After 15 days of exposure to 200 mM NaCl salinity, the removal of stress allowed only the belowground root system of *H. maritimum* to fully recover from salinity, showing also higher nitrate, hexoses, sucrose, proteins, glutamate and glutamine content in comparison with *H. vulgare*. Probably the wild species during the relief from salt stress was able to activate protection mechanisms, also stimulated by the high sodium concentration in the vacuoles, which allowed better growth conditions than those of cultivated species. In fact, *H. maritimum* plant roots had a high capacity to use stored carbohydrates for synthetizing free amino acids, among which amides, aspartate, glutamate, ornithine and threonine. This is in agreement with the results of de Lacerda, et al. [69], demonstrating that starch was depleted in sorghum roots during salt stress recovery and with those of Al-Hakimi and Hamada [70] proving that amelioration of salinity stress in bread wheat determined decrease of starch accumulation in roots. The released carbon could be used for the biosynthesis of compatible solutes (like amino acids) allowing the plant to mitigate the effects of stress and therefore increasing the chances of recovering after salt stress relief [71].

High appears to be related to an adequate partition of carbon between shoots and roots and to changes in absorption, transport and re-translocation of salts.8

This latter, in fact, can be synthetized from aspartate by mean of an aspartate aminotransferase (AAT) that is involved in the recycling of the carbon skeletons during ammonia absorption in roots [72]. Threonine plays a key role in the transduction of signals from receptors that sense phytohormones and environmental stresses, being able to translate them into specific functional outputs such as plant changes in gene expression and metabolism, cell growth and division, plant growth and abiotic stress tolerance [73]. The strong increase of ornithine is also noteworthy since it can function as precursor of polyamines via ornithine decarboxylase (ODC), metabolites that have been associated with increased resistance of plants to salt and drought stresses [74]. However, notwithstanding the better performance of *H. maritimum* in comparison with *H. vulgare* after stress removal, all the considered parameters did not reach values comparable to those of the cultivated species under control conditions.

## 4. Materials and Methods

### 4.1. Plant Material and Growth Conditions

Seeds of cultivated barley (*Hordeum vulgare* L. cv. Lamsi, 2*n* = 14) [8,75], provided by the National Agronomy Institutes of Tunisia (INAT) and seeds of wild barley *Hordeum maritimum* With. (*H. marinum* Huds. subsp. *marinum*, 2*n* = 14), collected from Kelbia Sebkha (an intermittent lake in Tunisia that covers 8000 ha in Sousse Governorate, at 35°50′34″ N, 10°16′18″ E south of Kondar) (Ferchichi et al. [43] and references therein) were used for the experiments. *H. marinum,* also known as seaside barley, is a true halophile species present in Tunisian saline depressions having a soluble Na^+^ content of about 90 µmol g^−1^ soil (ECe of 19.0 dS m^−1^), corresponding to about 200 mM [76]. In these salt-affected ecosystems, this species significantly contributes to annual biomass production and results very useful for fodder production, like sorghum in arid areas of Iran [77,78,79,80,81].

The seeds, previously disinfected with 1% NaOCl for 5 min and then watered with sterile distilled water, were germinated on two layers of Whatman filter paper moistened with sterile distilled water in the dark at 25 °C. The seeds of the two species had different latency time (the time after which no seed has yet germinated), which were 24 and 72 h for *H. vulgare* and *H. maritimum*, respectively. The total germination under dark was 80.8% and 68.8% for the cultivated and wild species, respectively (Figure S2). The delay and lower germination of *H. maritimum* seeds compared to *H. vulgare* one may be due to the exposure of mother plants to saline habitat during the development that can affect the germination attributes of seeds [82,83].

Five-day-old seedlings (at the same vegetative stage) were transferred into 5-L plastic pots with perforated plastic tops (20 plants per pot) containing aerated nutrient solution (Hewitt 1966), which was replaced every 2 days and grown under controlled conditions (16 h photoperiod, 350 µmol m^–2^s^–1^ PAR, thermoperiod 25/20 °C day/night, 65% RH). Treatments of the two cultivars of barley were arranged in a randomized design and, for each treatment, three replicate pots were used. On day 15 of hydroponic culture (days after sowing, DAS), 50 mM NaCl were added to the culture medium of two out of three treatments (salt stress and salt removal), each 12 h for two days up to 200mM NaCl. As reported by Shavrukov, et al. [84], the progressive exposure to NaCl stress prevents salt shock and reflects the field growing conditions. The control plants were grown without the addition of NaCl. Starting from 30 DAS, the plants of the salt removal treatments were grown without adding NaCl in the culture media. The plants, of each treatment, were harvested at 30, 33, 36, 42, 48 DAS and separated in shoots and roots. The measurements of fresh weight, relative water content, water potential and solutes potential were immediately performed, while another part of the plant material was shock frozen in liquid nitrogen and transported on dry ice to the laboratory of Plant Physiology of University of Campania “Luigi Vanvitelli,” where they were ground to a fine powder in liquid nitrogen and either used immediately for assays or stored at –80 °C.

### 4.2. Measurements of FW and DW, Water Relations and Osmolality

Plants were harvested 4 h after the beginning of the light period and divided into leaves and roots. Roots were immediately weighed to obtain the fresh weight (FW), reweighted after floating on deionized water for 24 h at 4 °C in the dark for obtaining the turgid weight (TW) and after being dried at 80 °C for a week for dry weight (DW) determination. Relative water content (RWC) was calculated according to Schonfeld, et al. [85]:

RWC (%) = ((FW–DW)/(TW–DW)) × 100(1)

Roots water potential (ψ_L_) was measured on five mature roots by Scholander pressure-chamber technique, 6–8 h after the onset of the light period (Scholander et al. 1965). For the measurement of the solute potential (ψs) freshly harvested roots were cut into small pieces, placed in Eppendorf tubes and crushed with a pestle before being centrifuged at 15,000× *g* for 15 min at 4 °C. The supernatant was collected to measure the osmolality of roots sap using a vapor pressure osmometer (Wescov 5500) [86].

### 4.3. Ions, Hydrogen Peroxide, Malondialdehyde and Metabolites Analysis

Ions were extracted from 50 mg samples of powdered dried roots in 5 mL of ultrapure water (Milli-Q PLUS, Millipore) and subjected to three freeze-thaw cycles according to Carillo, et al. [87]. After centrifugation, the clear supernatants were analyzed by ion-exchange chromatography using a DX500 apparatus (Dionex, Sunnyvale, CA, USA), an IONPAC-ATC1 anion trap column (Dionex), an IONPAC-AG11 guard column (Dionex) and an analytical IONPAC-AS11 4-mm column (Dionex), fitted with an ASRSII 4-mm suppressor for anions (Dionex) and an IONPAC-CTC cation trap column (Dionex), an IONPA-CCG12A guard column (Dionex) and an analytical IONPAC-CS12A 4-mm column (Dionex), fitted with a CSRS 4-mm suppressor for cations (Dionex), coupled to a CD20 conductivity detector (Dionex). Hydrogen peroxide (H_2_O_2_) content was determined according to Baptista, et al. [88] with few modifications. Aliquots of 40 mg of frozen powder roots were suspended in 1.0 mL of 0.1% (*w*/*v*) of trichloroacetic acid and, after centrifugation at 14,000× *g* at 4 °C for 10 min, 50 µL of clear supernatants or standard H_2_O_2_ (50 µL of 0.5, 1, 2 and 4 mM H_2_O_2_ corresponding to 25, 50, 100 and 200 nmoles in the well) were placed in wells of a polypropylene microplate with 50 µL of 10 mM phosphate buffer (pH 7.0) and 100 µL of 1M KI. H_2_O_2_ content was estimated at 390 nm for comparison with standard curves of known concentrations of H_2_O_2_. Malondialdehyde (MDA) was extracted by mixing 20 mg frozen roots samples with 1mL of ethanol:water (80:20, *v*/*v*), centrifuged at 14,000× *g* (5min) and successively analyzed according to [89]. Total proteins, starch and sugars were evaluated according to Carillo, et al. [90]. Starch was expressed as glucose equivalents. Proline, glycine betaine and primary amino acids, were extracted and assayed by FLD-HPLC according to Woodrow, Ciarmiello, Annunziata, Pacifico, Iannuzzi, Mirto, D’Amelia, Dell’Aversana, Piccolella, Fuggi and Carillo [6]. Contribution of metabolites and ions to osmolality was calculated according to Cuin, Tian, Betts, Chalmandrier and Shabala [86].

### 4.4. Statistical Analysis

Roots from five plants for each treatment were used for measurements of FW and DW, length, water potential and solute potential. All the other analyses were performed on three biological replicates for each treatment, each one constituted of 10 plant roots pooled together. The analysis of variance (ANOVA) and the Pearson correlation analysis were performed by SigmaPlot 12 software (Systat Software Inc., San Jose, CA, USA). The mean differences were compared with their corresponding Least Significant Differences (l.s.d.) at 0.05 confidence level. A principal component analysis (PCA) was conducted using Minitab 18.1 statistical software (Minitab Inc., Coventry, UK), according to Ciarmiello, et al. [91]. The PCA outputs included variable loading to each selected component and treatment component scores [37].

## 5. Conclusions

The results obtained provide important information on the different capacity of the two considered barley species to cope with the prolonged salt stress, thanks also to the integration of morphological, physiological and metabolic profile data. The roots of *H. maritimum* mostly used the compartmentalization of toxic ions into vacuoles as a defense mechanism. In *H. vulgare*, on the other hand, the synthesis of compatible osmolytes, particularly in the first weeks of salinity, was more active than other responses. In the last stages of stress, the wild species increased the root to shoot ratio to enhance the retention function of inorganic ions to avoid they could reach and damage shoot cells. However, this only entailed a delay of 2–3 weeks in the appearance of toxic effects. In fact, during prolonged salinization the smaller portion of shoot cannot provide sufficient assimilates from which to obtain energy to be used both for the transport of ions in the vacuole and for the synthesis of compatible solutes [9]. Nevertheless, these few weeks delay of the wild species in showing the symptoms of stress compared with the cultivated one may be pivotal for enabling the survival of plants when soil salinity is transient and not permanent [43]. Moreover, the protection mechanisms activated by *H. maritimum* allowed this species an interesting recovery of the growth parameters during short-term salinization, showing a better behavior than *H. vulgare*.

In summary, because of its wide distribution in most saline areas and major contribution in biomass production, *H. maritimum* has become an important model plant for physiologists and ecologists and has been studied extensively in contexts of competition, ecological succession, nitrogen availability and salinity gradients. *H. maritimum* also constitutes an extremely important plant genetic resource for genetic improvement programs. The insertion of specific genes into transgenic plants, such as those coding for ion transporters, compatible solutes and antioxidant molecules [33], is one of the main research approaches to improve the salinity tolerance of crop species of economic interest and pave the way for agricultural production in marginal and saline environments.

## Figures and Tables

**Table 1:** Relative contribution (%) of inorganic ions, amino acids, glycine betaine, soluble sugars and other metabolites towards the total osmolality of control and salt stressed roots of *Hordeum maritimum* (Table 1A) and *Hordeum vulgare* (Table 1B) at 30, 36 and 48 days after sowing (DAS). Salt was gradually added to salt treated plants starting from 15 DAS. Values are mean ± s.d. (*n* = 3). The s.d. was lower than 14.6% of the average value.

**A. *Hordeum maritimum***	**30 Days**		**36 Days**			**48 Days**		
	**C**	**S/R**	**C**	**S**	**R**	**C**	**S**	**R**
**Osmolarity (mOsmol/kg)**	**330**	**617**	**307**	**553**	**460**	**315**	**645**	**453**
Chloride	5.4	10.2	7.1	10.4	6.3	8.2	8.7	4.8
Nitrate	10.7	4.5	11.3	4.7	7.0	11.5	2.3	8.2
Potassium	17.8	7.2	20.1	5.8	10.1	20.1	3.1	12.4
Sodium	6.0	17.1	8.2	19.7	15.5	10.4	17.7	13.5
**Measured ions**	**39.9**	**39.0**	**46.6**	**40.6**	**38.9**	**50.3**	**31.8**	**38.9**
Hexoses	4.3	3.0	1.4	1.1	0.5	1.2	1.1	1.4
Sucrose	0.5	0.6	0.2	0.2	0.1	0.1	0.2	0.1
**Sum of Soluble Sugars**	**4.7**	**3.6**	**1.6**	**1.3**	**0.6**	**1.4**	**1.3**	**0.6**
**Total Amino Acids**	**14.4**	**7.8**	**20.5**	**6.7**	**8.1**	**16.6**	**3.4**	**13.4**
Asn	1.2	0.4	2.7	0.4	0.9	2.9	0.2	1.9
GABA	0.0	0.0	0.0	0.0	0.1	0.0	0.0	0.1
Gln	9.3	5.3	12.6	4.5	4.6	10.0	1.8	8.0
Glu	0.6	0.4	0.6	0.2	0.3	0.5	0.2	0.6
Pro	0.7	0.5	0.7	0.5	0.4	0.6	0.4	0.4
Minor AA	1.3	0.5	2.4	0.4	0.7	1.6	0.4	1.3
**Glycine betaine**	**0.2**	**0.2**	**0.3**	**0.2**	**0.1**	**0.2**	**0.1**	**0.1**
**Measured Organic Osmolytes**	**19.3**	**11.6**	**22.4**	**8.2**	**8.9**	**18.1**	**4.8**	**15.1**
**Other Metabolites**	**40.7**	**49.4**	**31.0**	**51.2**	**52.2**	**31.6**	**63.4**	**46.0**
**B. *Hordeum vulgare***	**30 Days**		**36 Days**			**48 Days**		
	**C**	**S/R**	**C**	**S**	**R**	**C**	**S**	**R**
**Osmolarity (mOsmol/kg)**	**354**	**370**	**338**	**393**	**304**	**295**	**475**	**292**
Chloride	4.0	15.0	4.7	15.8	16.7	4.9	13.6	6.0
Nitrate	10.5	6.5	10.3	4.7	9.5	10.0	1.6	10.4
Potassium	17.6	6.2	19.3	3.9	16.6	18.4	1.9	20.3
Sodium	7.5	11.8	7.2	15.6	21.7	8.6	17.3	8.3
**Measured ions**	**39.5**	**39.5**	**41.4**	**40.0**	**64.5**	**41.8**	**34.5**	**45.0**
Hexoses	2.3	1.3	4.9	5.4	5.8	7.6	1.8	6.1
Sucrose	0.3	0.5	0.5	0.7	0.7	1.1	0.2	0.5
**Sum of Soluble Sugars**	**2.7**	**1.8**	**5.4**	**6.1**	**6.6**	**8.7**	**2.00**	**6.6**
**Total Amino Acids**	**7.4**	**8.8**	**7.0**	**11.9**	**10.8**	**9.7**	**3.9**	**12.0**
Asn	0.3	0.9	0.2	0.7	0.6	0.4	0.3	0.8
GABA	0.1	0.0	0.3	0.1	0.1	0.3	0.0	0.3
Gln	3.8	4.6	2.7	7.4	6.2	5.1	1.5	6.7
Glu	0.8	0.5	1.0	0.7	0.9	0.6	0.2	0.5
Pro	0.5	0.6	0.6	0.7	0.7	0.7	0.5	0.7
Minor AA	0.9	1.0	0.9	1.0	0.8	1.3	0.7	1.6
**Glycine betaine**	**0.3**	**0.3**	**0.3**	**0.4**	**0.6**	**0.2**	**0.2**	**0.3**
**Measured Organic Osmolytes**	**10.3**	**10.8**	**12.7**	**18.5**	**17.9**	**18.6**	**6.2**	**18.9**
**Other Metabolites**	**50.1**	**49.6**	**45.9**	**41.5**	**17.6**	**39.6**	**59.4**	**36.1**

## Data Availability

Not applicable.

## Supplementary Materials

The following are available online at https://www.mdpi.com/2223-7747/10/2/307/s1, Figure S1: Plants’ photos, Figure S2: Germination percentage of seeds of *H. maritimum* and *H. vulgar*e, Table S1: All data.

## References

[1] Maggio A., De Pascale S., Fagnano M., Barbieri G. (2011). Saline agriculture in Mediterranean environments. Ital. J. Agron..

[2] Carillo P., Grazia M., Pontecorvo G., Fuggi A., Woodrow P. (2011). Salinity Stress and Salt Tolerance. Abiotic Stress in Plants—Mechanisms and Adaptations.

[3] Machado R.M.A., Serralheiro R.P. (2017). Soil Salinity: Effect on Vegetable Crop Growth. Management Practices to Prevent and Mitigate Soil Salinization. Horticulturae.

[4] Shrivastava P., Kumar R. (2015). Soil salinity: A serious environmental issue and plant growth promoting bacteria as one of the tools for its alleviation. Saudi J. Biol. Sci..

[5] Hessini K., Jeddi K., Siddique K.H.M., Cruz C. (2020). Drought and salinity: A comparison of their effects on the ammoni-um-preferring species Spartina alterniflora. Physiol. Plant..

[6] Woodrow P., Ciarmiello L.F., Annunziata M.G., Pacifico S., Iannuzzi F., Mirto A., D’Amelia L., Dell’Aversana E., Piccolella S., Fuggi A. (2016). Durum wheat seedling responses to simultaneous high light and salinity involve a fine reconfiguration of amino acids and carbohydrate metabolism. Physiol. Plant..

[7] Hasanuzzaman M., Nahar K., Alam M., Bhowmik P.C., Hossain A., Rahman M.M., Prasad M.N.V., Ozturk M., Fujita M. (2014). Potential Use of Halophytes to Remediate Saline Soils. BioMed Res. Int..

[8] Hessini K., Ferchichi S., Ben Youssef S., Werner K.H., Cruz C., Gandour M. (2015). How Does Salinity Duration Affect Growth and Productivity of Cultivated Barley?. Agron. J..

[9] Flowers T.J., Munns R., Colmer T.D. (2015). Sodium chloride toxicity and the cellular basis of salt tolerance in halophytes. Ann. Bot..

[10] Shabala S., Munns R. (2012). Salinity Stress: Physiological Constraints and Adaptive Mechanisms.

[11] Carillo P., Cirillo C., De Micco V., Arena C., De Pascale S., Rouphael Y. (2019). Morpho-anatomical, physiological and biochemical adaptive responses to saline water of Bougainvillea spectabilis Willd. trained to different canopy shapes. Agric. Water Manag..

[12] Neumann P.M., Baluška F., Čiamporová M., Gašparíková O., Barlow P.W. (1995). Inhibition of root growth by salinity stress: Toxicity or an adaptive biophysical response?. Structure and Function of Roots, Proceedings of the Fourth International Symposium on Structure and Function of Roots, Stará Lesná, Slovakia, 20–26 June 1993.

[13] Cramer G.R., Läuchli A., Polito V.S. (1985). Displacement of Ca^2+^ by Na^+^ from the Plasmalemma of Root Cells: A Primary Response to Salt Stress?. Plant Physiol..

[14] Flowers T.J., Hajibagheri M.A. (2001). Salinity tolerance in Hordeum vulgare: Ion concentrations in root cells of cultivars differing in salt tolerance**. Plant Soil.

[15] Shabala S. (2013). Learning from halophytes: Physiological basis and strategies to improve abiotic stress tolerance in crops. Ann. Bot..

[16] Ciarmiello L.F., Woodrow P., Piccirillo P., De Luca A., Carillo P. (2014). Transcription Factors and Environmental Stresses in Plants. Emerging Technologies and Management of Crop Stress Tolerance.

[17] Woodrow P., Pontecorvo G., Fantaccione S., Fuggi A., Kafantaris I., Parisi D., Carillo P. (2010). Polymorphism of a new Ty1-copia retrotransposon in durum wheat under salt and light stresses. Theor. Appl. Genet..

[18] Woodrow P., Pontecorvo G., Ciarmiello L.F., Fuggi A., Carillo P. (2011). Ttd1a promoter is involved in DNA–protein binding by salt and light stresses. Mol. Biol. Rep..

[19] James R.A., Blake C., Byrt C.S., Munns R. (2011). Major genes for Na^+^ exclusion, Nax1 and Nax2 (wheat HKT1;4 and HKT1;5), decrease Na+ accumulation in bread wheat leaves under saline and waterlogged conditions. J. Exp. Bot..

[20] Carillo P., Woodrow P., Raimondi G., El-Nakhel C., Pannico A., Kyriacou M.C., Colla G., Mori M., Giordano M., Giordano M. (2019). Omeprazole Promotes Chloride Exclusion and Induces Salt Tolerance in Greenhouse Basil. Agronomy.

[21] Yancey P.H. (2005). Organic osmolytes as compatible, metabolic and counteracting cytoprotectants in high osmolarity and other stresses. J. Exp. Biol..

[22] Puniran-Hartley N., Hartley J., Shabala L., Shabala S. (2014). Salinity-induced accumulation of organic osmolytes in barley and wheat leaves correlates with increased oxidative stress tolerance: In planta evidence for cross-tolerance. Plant Physiol. Biochem..

[23] Carillo P., Mastrolonardo G., Nacca F., Parisi D., Verlotta A., Fuggi A. (2008). Nitrogen metabolism in durum wheat under salinity: Accumulation of proline and glycine betaine. Funct. Plant Biol..

[24] Mansour M.M. (2000). Nitrogen Containing Compounds and Adaptation of Plants to Salinity Stress. Biol. Plant..

[25] Suprasanna P., Nikalje G.C., Rai A.N., Iqbal N., Nazar R.A., Khan N. (2016). Osmolyte Accumulation and Implications in Plant Abiotic Stress Tolerance. Osmolytes and Plants Acclimation to Changing Environment: Emerging Omics Technologies.

[26] Annunziata M.G., Ciarmiello L.F., Woodrow P., Dell’Aversana E., Carillo P. (2019). Spatial and Temporal Profile of Glycine Betaine Accumulation in Plants Under Abiotic Stresses. Front. Plant Sci..

[27] Negrão S., Schmöckel S.M., Tester M. (2017). Evaluating physiological responses of plants to salinity stress. Ann. Bot..

[28] Kumar D., Al Hassan M., Naranjo M.A., Agrawal V., Boscaiu M., Vicente O. (2017). Effects of salinity and drought on growth, ionic relations, compatible solutes and activation of antioxidant systems in oleander (Nerium oleander L.). PLoS ONE.

[29] Maas E.V., Nieman R.H., Jung G.A. (2015). Physiology of Plant Tolerance to Salinity. Soil Erosion and Conservation in the Tropics.

[30] Stoskopf N.C. (1985). Cereal Grain Crops.

[31] Nongpiur R.C., Singla-Pareek S.L., Pareek A. (2016). Genomics Approaches for Improving Salinity Stress Tolerance in Crop Plants. Curr. Genom..

[32] Vij S., Tyagi A.K. (2007). Emerging trends in the functional genomics of the abiotic stress response in crop plants. Plant Biotechnol. J..

[33] Budak H., Hussain B., Khan Z., Ozturk N.Z., Ullah N. (2015). From Genetics to Functional Genomics: Improvement in Drought Signaling and Tolerance in Wheat. Front. Plant Sci..

[34] Yang Y., Guo Y. (2017). Elucidating the molecular mechanisms mediating plant salt-stress responses. New Phytol..

[35] Costa S.F., Martins D., Agacka-Mołdoch M., Czubacka A., Araújo S.D.S. (2018). Strategies to Alleviate Salinity Stress in Plants. Salinity Responses and Tolerance in Plants.

[36] Cuevas J., Daliakopoulos I.N., Del Moral F., Hueso J.J., Tsanis I. (2019). A Review of Soil-Improving Cropping Systems for Soil Salinization. Agronomy.

[37] Rouphael Y., Raimondi G., Lucini L., Carillo P., Kyriacou M.C., Colla G., Cirillo V., Pannico A., El-Nakhel C., De De Pascale S. (2018). Physiological and Metabolic Responses Triggered by Omeprazole Improve Tomato Plant Tolerance to NaCl Stress. Front. Plant Sci..

[38] Carillo P., Ciarmiello L.F., Woodrow P., Corrado G., Chiaiese P., Rouphael Y. (2020). Enhancing Sustainability by Improving Plant Salt Tolerance through Macro- and Micro-Algal Biostimulants. Biology.

[39] Letey J., Hoffman G., Hopmans J.W., Grattan S., Suarez D.L., Corwin D.L., Oster J., Wu L., Amrhein C. (2011). Evaluation of soil salinity leaching requirement guidelines. Agric. Water Manag..

[40] Evolkov V. (2015). Salinity tolerance in plants. Quantitative approach to ion transport starting from halophytes and stepping to genetic and protein engineering for manipulating ion fluxes. Front. Plant Sci..

[41] Yousefi A.R., Rashidi S., Moradi P., Mastinu A. (2020). Germination and Seedling Growth Responses of Zygophyllum fabago, Salsola kali L. and Atriplex canescens to PEG-Induced Drought Stress. Environments.

[42] Munns R., James R.A., Läuchli A. (2006). Approaches to increasing the salt tolerance of wheat and other cereals. J. Exp. Bot..

[43] Ferchichi S., Hessini K., Dell’Aversana E., D’Amelia L., Woodrow P., Ciarmiello L.F., Fuggi A., Carillo P. (2018). Hordeum vulgare and Hordeum maritimum respond to extended salinity stress displaying different temporal accumulation pattern of metabolites. Funct. Plant Biol..

[44] Garthwaite A.J., Von Bothmer R., Colmer T.D. (2005). Salt tolerance in wild Hordeum species is associated with restricted entry of Na^+^ and Cl^−^ into the shoots. J. Exp. Bot..

[45] Gorham J., Jones R.G.W., Bristol A. (1990). Partial characterization of the trait for enhanced K+−Na+ discrimination in the D genome of wheat. Planta.

[46] EAbdElGawad H., Ezinta G., Hegab M.M., Epandey R., Easard H., Eabuelsoud W. (2016). High Salinity Induces Different Oxidative Stress and Antioxidant Responses in Maize Seedlings Organs. Front. Plant Sci..

[47] Hessini K., Martínez J.P., Gandour M., Albouchi A., Soltani A., Abdelly C. (2009). Effect of water stress on growth, osmotic adjustment, cell wall elasticity and water-use efficiency in Spartina alterniflora. Environ. Exp. Bot..

[48] Acosta-Motos J.R., Ortuño M.F., Bernal-Vicente A., Diaz-Vivancos P., Sanchez-Blanco M.J., Hernandez J.A. (2017). Plant Responses to Salt Stress: Adaptive Mechanisms. Agronomy.

[49] James R.A., Von Caemmerer S., Condon A.G., Zwart A.B., Munns R. (2008). Genetic variation in tolerance to the osmotic stress componentof salinity stress in durum wheat. Funct. Plant Biol..

[50] Annunziata M.G., Ciarmiello L.F., Woodrow P., Maximova E., Fuggi A., Carillo P. (2017). Durum Wheat Roots Adapt to Salinity Remodeling the Cellular Content of Nitrogen Metabolites and Sucrose. Front. Plant Sci..

[51] Rahnama A., Munns R., Poustini K., Watt M. (2011). A screening method to identify genetic variation in root growth response to a salinity gradient. J. Exp. Bot..

[52] Deinlein U., Stephan A.B., Horie T., Luo W., Xu G., Schroeder J.I. (2014). Plant salt-tolerance mechanisms. Trends Plant Sci..

[53] Keys A.J. (2006). The re-assimilation of ammonia produced by photorespiration and the nitrogen economy of C3 higher plants. Photosynth. Res..

[54] Abogadallah G.M., Serag M.S., Quick W.P. (2010). Fine and coarse regulation of reactive oxygen species in the salt tolerant mutants of barnyard grass and their wild-type parents under salt stress. Physiol. Plant..

[55] Ma J., Cirillo V., Zhang D., Zhang D., Wang L., Xiao X., Yao Y. (2020). Regulation of Ammonium Cellular Levels is An Important Adaptive Trait for the Euhalophytic Behavior of Salicornia europaea. Plants.

[56] Rozema J., Bijwaard P., Prast G., Broekman R. (1985). Ecophysiological adaptations of coastal halophytes from foredunes and salt marshes. Vegetatio.

[57] MacNeill G.J., Mehrpouyan S.A.A., Minow M.A., Patterson J., Tetlow I.J., Emes M.J. (2017). Starch as a source, starch as a sink: The bifunctional role of starch in carbon allocation. J. Exp. Bot..

[58] Carillo P. (2018). GABA Shunt in Durum Wheat. Front. Plant Sci..

[59] Yousfi S., Rabhi M., Hessini K., Abdelly C., Gharsalli M. (2009). Differences in efficient metabolite management and nutrient metabolic regulation between wild and cultivated barley grown at high salinity. Plant Biol..

[60] Li W., Wang D., Jin T., Chang Q., Yin D., Xu S., Liu B., Liu L. (2010). The Vacuolar Na^+^/H^+^ Antiporter Gene SsNHX1 from the Halophyte Salsola soda Confers Salt Tolerance in Transgenic Alfalfa (Medicago sativa L.). Plant Mol. Biol. Rep..

[61] Garbarino J., Dupont F.M. (1988). NaCl Induces a Na^+^/H^+^ Antiport in Tonoplast Vesicles from Barley Roots. Plant Physiol..

[62] Al-Yasi H., Attia H., Alamer K., Hassan F., Ali E., Elshazly S., Siddique K.H., Hessini K., Esmat F. (2020). Impact of drought on growth, photosynthesis, osmotic adjustment, and cell wall elasticity in Damask rose. Plant Physiol. Biochem..

[63] Rubio F., Gassmann W., Schroeder J.I. (1995). Sodium-Driven Potassium Uptake by the Plant Potassium Transporter HKT1 and Mutations Conferring Salt Tolerance. Science.

[64] Assaha D.V.M., Ueda A., Saneoka H., Al-Yahyai R., Yaish M.W. (2017). The role of Na^+^ and K^+^ transporters in salt stress adaptation in glycophytes. Front. Physiol..

[65] Ligaba A., Katsuhara M. (2009). Insights into the salt tolerance mechanism in barley (Hordeum vulgare) from comparisons of cultivars that differ in salt sensitivity. J. Plant Res..

[66] Bose J., Munns R., Shabala S., Gilliham M., Pogson B., Tyerman S. (2017). Chloroplast function and ion regulation in plants growing on saline soils: Lessons from halophytes. J. Exp. Bot..

[67] Wang M., Zheng Q., Shen Q., Guo S. (2013). The Critical Role of Potassium in Plant Stress Response. Int. J. Mol. Sci..

[68] Hsiao T.C., Xu L. (2000). Sensitivity of growth of roots versus leaves to water stress: Biophysical analysis and relation to water transport. J. Exp. Bot..

[69] De Lacerda C.F., Cambraia J., Oliva M.A., Ruiz H.A. (2005). Changes in growth and in solute concentrations in sorghum leaves and roots during salt stress recovery. Environ. Exp. Bot..

[70] Al-Hakimi A., Hamada A. (2001). Counteraction of Salinity Stress on Wheat Plants by Grain Soaking in Ascorbic Acid, Thiamin or Sodium Salicylate. Biol. Plant..

[71] Thalmann M., Santelia D. (2017). Starch as a determinant of plant fitness under abiotic stress. New Phytol..

[72] Ryan E., Fottrell P.F. (1974). Subcellular localization of enzymes involved in the assimilation of Ammonia by soybean root nodules. Phytochemistry.

[73] Muthuramalingam P., Krishnan S.R., Pandian S., Mareeswaran N., Aruni W., Pandian S.K., Ramesh M. (2018). Global analysis of threonine metabolism genes unravel key players in rice to improve the abiotic stress tolerance. Sci. Rep..

[74] Kalamaki M.S., Merkouropoulos G., Kanellis A.K. (2009). Can ornithine accumulation modulate abiotic stress tolerance in Arabidopsis?. Plant Signal. Behav..

[75] Mejri M., Siddique K.H.M., Saif T., Abdelly C., Hessini K. (2016). Comparative effect of drought duration on growth, photosynthesis, water relations, and solute accumulation in wild and cultivated barley species. J. Plant Nutr. Soil Sci..

[76] Rabhi M., Hafsi C., Lakhdar A., Barhoumi Z., Abdelly C., Smaoui A. (2009). Evaluation of the capacity of three halophytes to desalinize their rhizosphere as grown on saline soils under nonleaching conditions. Afr. J. Ecol..

[77] Lombardi T., Lupi B. (2006). Salt-tolerance in wild *Hordeum* (Poaceae) species: Differences between *H. maritimum* With. and *H. hystrix Roth*. Atti. Soc. Tosc. Sci. Nat..

[78] Hafsi C., Lakhdhar A., Rabhi M., Debez A., Abdelly C., Ouerghi Z. (2007). Interactive effects of salinity and potassium availability on growth, water status, and ionic composition of Hordeum maritimum. J. Plant Nutr. Soil Sci..

[79] Hammami H., Gomes T., Abdelly C., Mahmoud O.M.-B., Baptista P., Martins F. (2016). Impact of a natural soil salinity gradient on fungal endophytes in wild barley (Hordeum maritimum With.). World J. Microbiol. Biotechnol..

[80] Abdelly C., Lachaal M., Grignon C., Soltani A., Hajji M. (1995). Association épisodique d’halophytes strictes et de glycophytes dans un écosystème hydromorphe salé en zone semi-aride. Agronomie.

[81] Rad S.V., Valadabadi S.A.R., Pouryousef M., Saifzadeh S., Zakrin H.R., Mastinu A. (2020). Quantitative and Qualitative Evaluation of Sorghum bicolor L. under Intercropping with Legumes and Different Weed Control Methods. Horticulturae.

[82] El-Keblawy A., Al-Shamsi N., Mosa K. (2018). Effect of maternal habitat, temperature and light on germination and salt tolerance of Suaeda vermiculata, a habitat-indifferent halophyte of arid Arabian deserts. Seed Sci. Res..

[83] Shah S.Z., Rasheed A., Gul B., Khan M.A., Nielsen B.L., Hameed A. (2020). Maternal salinity improves yield, size and stress tolerance of Suaeda fruticosa seeds. J. Arid. Land.

[84] Shavrukov Y., Bovill J., Afzal I., Hayes J.E., Roy S.J., Tester M., Collins N.C. (2013). HVP10 encoding V-PPase is a prime candidate for the barley HvNax3 sodium exclusion gene: Evidence from fine mapping and expression analysis. Planta.

[85] Schonfeld M.A., Johnson R.C., Carver B.F., Mornhinweg D.W. (1988). Water Relations in Winter Wheat as Drought Resistance Indicators. Crop. Sci..

[86] Cuin T.A., Tian Y., Betts S.A., Chalmandrier R., Shabala S. (2009). Ionic relations and osmotic adjustment in durum and bread wheat under saline conditions. Funct. Plant Biol..

[87] Carillo P., Parisi D., Woodrow P., Pontecorvo G., Massaro G., Annunziata M.G., Fuggi A., Sulpice R. (2011). Salt-induced accumulation of glycine betaine is inhibited by high light in durum wheat. Funct. Plant Biol..

[88] Baptista P., Martins A., Pais M.S., Tavares R.M., Lino-Neto T. (2007). Involvement of reactive oxygen species during early stages of ectomycorrhiza establishment between Castanea sativa and Pisolithus tinctorius. Mycorrhiza.

[89] Havaux M., Eymery F., Porfirova S., Rey P., Dörmann P. (2005). Vitamin E Protects against Photoinhibition and Photooxidative Stress in Arabidopsis thaliana. Plant Cell.

[90] Carillo P., Cacace D., De Pascale S., Rapacciuolo M., Fuggi A. (2012). Organic vs. traditional potato powder. Food Chem..

[91] Ciarmiello L.F., Piccirillo P., Carillo P., De Luca A., Woodrow P. (2015). Determination of the genetic relatedness of fig (Ficus carica L.) accessions using RAPD fingerprint and their agro-morphological characterization. S. Afr. J. Bot..

