# Supplementary material for: Salinity Duration Differently Modulates Physiological Parameters and Metabolites Profile in Roots of Two Contrasting Barley Genotypes
Source: Plants (Basel). 2021 Feb 5;10(2):307. doi: 10.3390/plants10020307 (PMC7914899; doi:10.3390/plants10020307)
Supplement: Supplementary file 1 [file plants-10-00307-s001.zip › Supplemental figures 1 and 2.docx]

Supplementary data


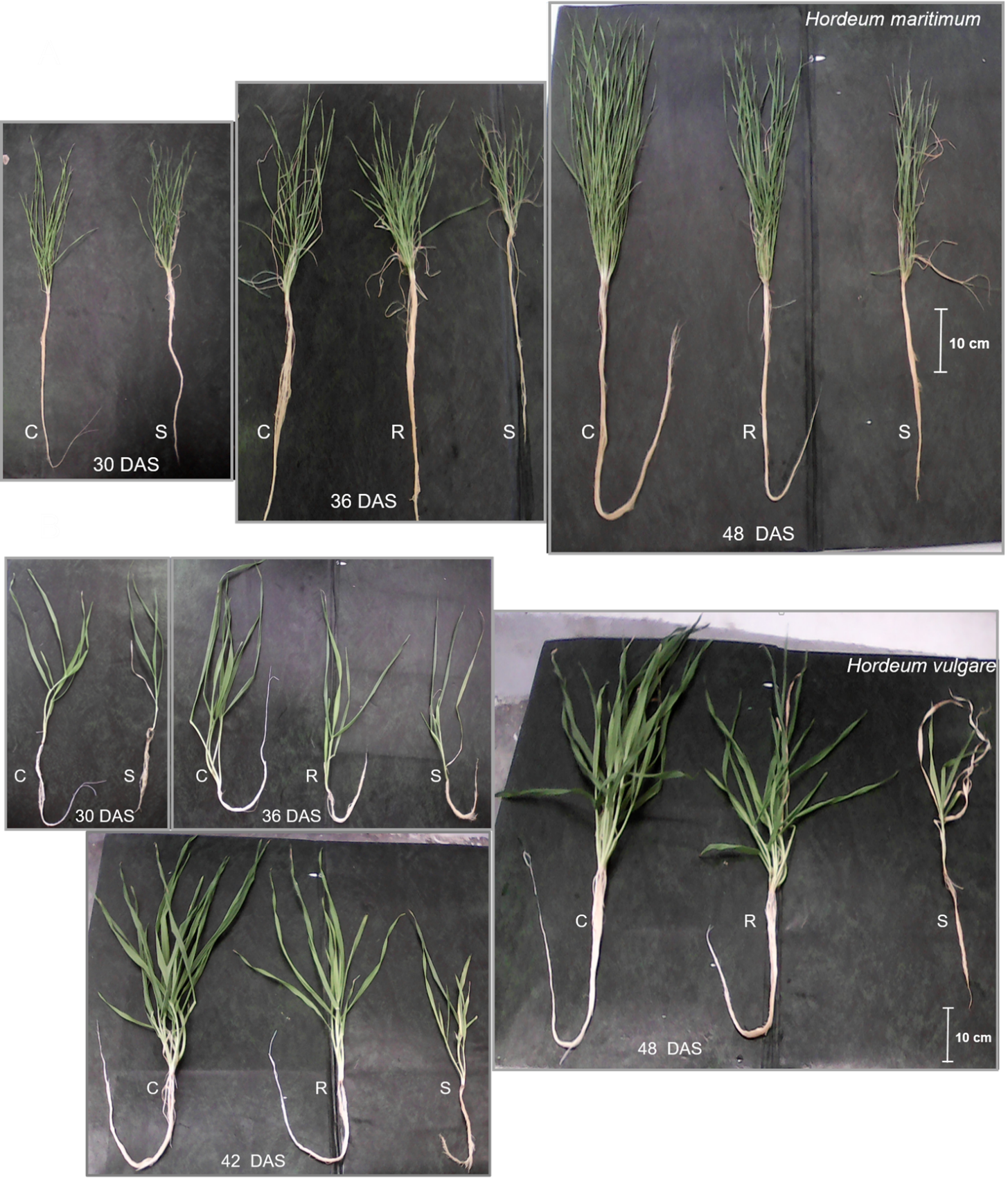


**Figure S1.** Effect of control, salt stress and salt removal treatments on phenotypic changes of *H. maritimum* and *H. vulgare*. Salt was gradually added to salinity treatments starting from 15 days after sowing (DAS). Harvests were conducted at 30, 33, 36, 42 and 48 DAS.


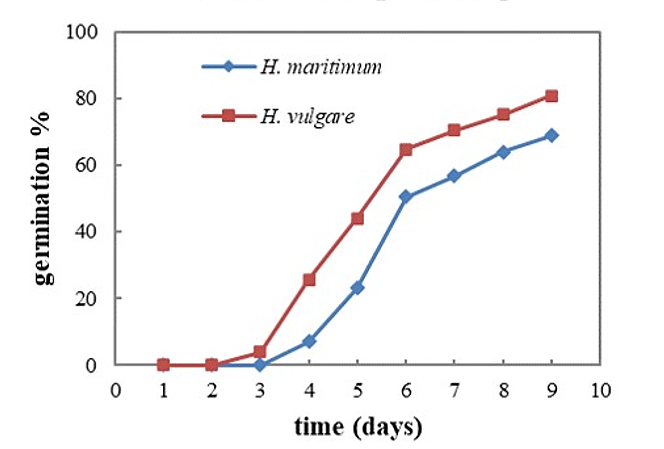


**Figure S2.** Germination percentage of seeds of *H. maritimum* and *H. vulgare*. The seeds, previously disinfected with 1% NaOCl for 5 min and then watered with sterile distilled water, were germinated on two layers of Whatman filter paper moistened with sterile distilled water in the dark at 25 °C.
